# The Effect of Education on Knowledge Regarding Breast Cancer Related Lymphedema Risk Reduction and Prevention Among Nursing Personnel

**DOI:** 10.7759/cureus.45331

**Published:** 2023-09-15

**Authors:** Malarvizhi K Natarajan, Nalini S J, Jaya Mohanraj, Usha Vishwanath

**Affiliations:** 1 Cardiovascular Nursing, HOSMAT College of Nursing, Bangalore, IND; 2 Obstetrics and Gynaecology Nursing, Sri Ramachandra Institute of Higher Education and Research, Chennai, IND; 3 Community Health Nursing, HOSMAT College of Nursing, Bangalore, IND; 4 Obstetrics and Gynaecology, Sri Ramachandra Institute of Higher Education and Research, Chennai, IND

**Keywords:** patient education, breast-cancer, risk reduction and prevention, nurse's knowledge, breast cancer-related lymphedema

## Abstract

Aim

The development of lymphedema post-breast-cancer surgery has been identified as a significant burden worldwide, with nurses at the forefront of prevention and risk reduction practices. Prevention is of crucial importance to avoid lymphedema formation and its complications. This study aims to assess the knowledge gained through an educational session regarding risk reduction and prevention of breast cancer-related lymphedema (BCRL) among nursing professionals and compare the pre-test and post-test knowledge.

Methods and material

The research approach was quantitative in nature, and the design adopted was a pre-experimental, one-group pre-test post-test design. The study was conducted in a 400-bed multispecialty teaching hospital in Bangalore, Karnataka. After obtaining formal permission from the authorities, the participants were approached and informed about the purpose of the study. Eighty-four staff nurses working with breast cancer-related lymphedema (BCRL) patients participated in the study. The data for the study was collected using a validated questionnaire based on the National Lymphedema Network's (NLN) breast cancer-related lymphedema (BCRL) risk reduction and preventive guidelines. The questionnaire consisted of two sections. Section A consisted of the staff nurses' demographic data, and Section B consisted of questions on risk reduction and prevention of lymphedema. The pre-test was conducted, followed by a structured teaching session on risk reduction and prevention of lymphedema among 84 staff nurses working with BCRL patients. After the teaching session, the post-test was conducted.

Results

Descriptive and inferential statistics were used for analysis in this study. The t-test determined the statistical significance using the software SPSS (SPSS Inc. Released 2007. SPSS for Windows, Version 16.0. Chicago, SPSS Inc.). The result showed that the mean knowledge score was 4.286 with SD 0.97 in the pre-test, which increased to 4.452 with SD 1.511 with a significant p-value (<0.001).

Conclusion

According to the study's findings, nurses must get standardized lymphedema training in order to prevent lymphedema from developing in patients having breast cancer surgery. The study's outcome has implications on the focus areas for nurses in the context of the team's adoption and dissemination of breast cancer-related lymphedema preventive measures.

Key messages of this study are - 1) BCRL is an irreversible, progressive complication with no cure if not diagnosed early. 2) Poor knowledge of lymphedema prevention among nurses leads to frustration for BCRL patients. 3) Risk reduction and prevention education enable the patient to reduce BCRL complications, minimize the severity of the condition, and improve the quality of life. 4) Pre-habilitation - patient education on early diagnosis and risk reduction/prevention of BCRL reduces the cost, time, and energy for the patient and health care delivery system.

## Introduction

Women treated for breast cancer commonly experience breast cancer-related lymphedema (BCRL) as a complication. Since lymphedema (LE) has no known treatment, vigilance and prevention are crucial to reduce mortality and lessen the severity of its long-term effects. It can be avoided or diminished with the appropriate nursing measures. In order to prevent LE, nurses are essential in assisting patients in accepting accountability for their own LE [1]. Patients' chances of acquiring breast cancer-related lymphedema (BCRL) can be lowered, and the disorder can be minimized by educating them about it.

Background

One of the studies claims that most medical professionals cannot adequately inform patients who are having breast surgery. According to studies, most patients are not given even the most basic information concerning complications of lymphedema (LE). Healthcare professionals (HCPs) must have specialized training in lymphedema physiology, risk reduction, and preventive strategies. By educating patients on the causes, signs, and symptoms of lymphedema, providing ongoing, thorough assessments of lymphedema during each visit, and supporting research in this area, nurses being on the front lines may hold the answer to actively avoiding lymphedema in many patients [2].

HCPs must have sufficient clinical knowledge to inform patients about breast cancer-related lymphedema (BCRL) appropriately. According to Thomas-MacLean et al., a lack of knowledge among HCPs is common and evident across various disciplines, including the surgeon, oncology department staff, and family physician [3]. Many patients believe that HCPs, including breast care nurses, surgeons, and family physicians, lack appropriate knowledge about lymphedema; consequently, these patients seek help from books, the internet, and self-help groups [4]. An essential part of the care of breast cancer survivors is providing advice on precautions and lifestyle modification to reduce the lifelong risk of developing BCRL [5]. HCPs should provide breast cancer patients with written and oral information at every follow-up appointment to help reduce the risk of developing lymphedema. Compared to patients who did not get information, those who did fare better with BCRL. Patients who felt they were not given enough information thought doctors underestimated the risk of BCRL; some wished their physicians had provided more information on how to prevent it, and many wished they had been made aware of the risks prior to surgery or at discharge because they thought BCRL was preventable. HCPs are not well-informed about lymphedema; thus, those who work closely with breast cancer patients need to know about the probable causes and risk factors for BCRL [6]. The required diversity of lymphedema education for healthcare professionals is demonstrated by the involvement of practitioners, including primary care doctors and surgeons, oncologists, nurses, physiotherapists, psychiatrists, dermatologists, and other specialists involved in lymphedema management [7].

Purpose of the study

HCPs understanding of lymphedema is crucial for educating patients or communities at risk. Finding the knowledge gaps in HCPs that may present an opportunity for appropriate interventions is crucial because lymphedema is not adequately addressed in medical research and education and may already have fallen by the wayside of healthcare delivery in various countries [8].

This study aims to assess the efficacy of education in raising nurses' awareness of the risk factors and preventive aspects of lymphedema among patients who underwent surgery for breast cancer. This information may help nurses better fulfill their duty to educate patients effectively, provide high-quality care, and improve health outcomes for those who are at risk for lymphedema.

Risk factors of breast cancer-related lymphedema

One of the most worrisome side effects of cancer treatment is lymphedema, which develops when protein-rich lymph fluid builds up in the subcutaneous tissue and the lymphatic system's carrying capacity is reduced [9]. LE can occur in cancer patients, especially after lymphadenectomy, adjuvant radiation, or in conjunction with major clinical risk factors [10]. Radiation therapy, sentinel node biopsy (5.6%), the type of breast surgery, and the level of axillary node involvement (19.9%) are all lifelong risk factors for post-breast cancer lymphedema (overall 21.4%). These factors reduce lymphatic outflow and promote fluid stasis in parts of the skin and subcutaneous tissues that drain to local lymph nodes [11]. Lymphedema is accompanied by a number of unpleasant symptoms, including swelling, pain, numbness, tightness, heaviness, and decreased limb mobility [12]. It results in physical and psychological issues for people, is worse by ongoing, progressive infections, frequently necessitates hospitalization, and is potentially lethal if not treated properly [1,10,13,14]. Additionally, this disease has an effect on quality of life (QoL), increasing the likelihood of side effects like cellulitis, obesity, skin abnormalities, and cancer susceptibility [15,16].

According to Torres et al., there are no nursing practice guidelines for educating about, assessing, managing, or monitoring lymphedema. For at least a year following surgery, educating patients about lymphedema "may help prevent and decrease subsequent lymphedema in patients undergoing breast cancer surgery, including dissection of axillary lymph nodes" [17]. The greatest hope for minimizing and controlling this pervasive illness in people who have received breast cancer treatment is early detection and intervention [18].

Nurses must be aware of this challenging issue in order to inform breast cancer patients about the lifelong dangers of contracting this illness. Breast cancer survivors should also be taught how to maintain a self-care routine for managing symptoms and preventing exacerbation in order to reduce their risk of developing lymphedema. The self-care plan calls for regular skin care, the use of gloves during daily activities to prevent skin breaks, protection from harm on the affected side, prevention of muscular strain, and lymphatic drainage promotion. During hospital stays related to breast cancer treatment, nurses should look for opportunities to instruct patients on lowering their risk of developing lymphedema or managing it. The recovery of the patient may be affected by such a strategy. Additionally, the same needs to be emphasized in the outpatient setting during survival [19].

Breast cancer-related lymphedema treatment

The benchmark for managing arm LE is complete decongestive therapy (CDT) performed by a certified lymphedema therapist or trained lymphedema physiotherapist or under the supervision of a trained lymphedema nurse [20,21]. The desired outcome of CDT is to move lymphatic fluid to a part or region where it can drain and help to lessen arm edema. Manual lymphatic drainage, short-stretch compression bandages and clothing, consistent arm workouts, and thorough skincare and hygiene are all part of CDT [22]. The patients are required to perform arm exercises on a set timetable that is rigid, regulated, and self-motivating. In order to detect the onset of cellulitis, promote quick treatment, and stop further damage of lymphatic transport, the patient and family must also be aware of the symptoms of acute inflammation.

Awareness and preventive action are crucial because it can be too late to avoid once symptoms appear. Women must be informed, educated, and instructed on preventative measures and cautionary behaviors to avoid lymphedema. They should start learning about lymphedema when choosing their breast cancer treatment.

According to studies, the majority of cancer patients are unaware of LE. This could be because the nurses do not consider patient education part of their job description or lack the necessary expertise [6,15,23,24]. When education is neglected, [16,24] patients' ability to manage this risk becomes insufficient. Additionally, patients' self-esteem and quality of life will improve by being taught how to prevent LE [16,25]. To successfully educate patients about LE, it is crucial to establish the notion that nurses have sufficient clinical skills and that patient education is important after cancer surgery [23].

Only a few studies look at nurses' knowledge, attitudes, and behaviors to avoid LE, even though a significant trend nowadays emphasizes the necessity of LE patient education. Preventing LE, a severe issue following cancer surgery should be a priority for nurses when educating patients. If nurses are not knowledgeable enough about lymphedema, they will not stress the importance of prevention or instruct patients on how to do so. The purpose of this study was to evaluate nurses' knowledge of LE.

By describing and documenting the effects of an education program on healthcare providers' understanding of the risk and prevention of lymphedema for women who have undergone breast cancer treatment, this study considerably contributes to the field of nursing. The study's findings might be applied to enrich the healthcare workers' training programs that work with this patient population. 

Research question

Will nurses attending educational sessions on breast cancer-related lymphedema show a greater understanding of the causes, symptoms, risk reduction, and prevention of lymphedema?

## Materials and methods

Study design

This study is a pre-experimental design that uses non-probability convenience sampling to examine the impact of an educational program on nurses' knowledge of reducing the risk and preventing breast cancer-related lymphedema among breast cancer patients.

Study population

The sample consisted of 84 female staff nurses in a teaching hospital eligible to care for patients undergoing post-mastectomy surgery for breast cancer treatment. The following factors were considered for sample selection for the study.

1. Nursing staff who are working in the selected hospital

2. Nursing staff who are available to attend the BCRL risk reduction and prevention session completely

3. Nursing staff working in oncology and post-operative wards

 

Data collection tool

Demographic Data

Questions one to six included age, gender, years of experience, professional qualification, and experience in the oncology department.

Knowledge Questionnaire

This included questions on risk reduction/prevention of BCRL based on the National Lymphedema Network (NLN). NLN is a position paper that provides information on lymphedema, its treatment, and risk reduction practices as guidelines for healthcare professionals and patients. The tool had two sections:

Sections A and B

Section A included 10 items consisting of general information on lymphedema and Section B included 33 items regarding the knowledge that nurses should impart to breast cancer patients in order to lower their chances of developing lymphedema (26 items) as well as (seven items) on measures to improve the dissemination of reducing the risk of BCRL.

Scoring and Interpretation

Each correct answer was scored as one and the wrong answer as zero. The total score was 43.

The score was interpreted as follows:

>75 % Highly adequate knowledge

50% - 75 % Moderately adequate knowledge

< 50% Inadequate Knowledge

Data collection 

The opinions of ten professionals, including faculty members, physicians, and nurses, were incorporated to ensure the questionnaire's content validity. These were further reviewed, and following the necessary preparations, ten nurses received pre-testing. The primary study did not include these nurses. The tool reliability was obtained by using a test-retest method, and the score was 0.86. The tool was reliable for conducting the study.

The 'convenience sampling' technique was used. Nurses who were present on the day of the educational session and ready to attend the session were included in the study.

After receiving approval from the Institution's Research Ethics Committee (HHEI/HCON/S-5/04/2021; HOSMAT College of Nursing, HOSMAT Hospital and Educational Institute), a one-group pre-test post-test design was conducted. The participants received a verbal and written explanation of the study's objectives prior to the collection of data. The right to secrecy and anonymity, the opportunity to contact the researcher with any queries, and the right to withdraw from the study were all guaranteed by the written informed consent given to each participant. The participant's agreement to participate was indicated by the completed paperwork being returned.

Eighty-four nurses in the study signed the consent form and received the questionnaire. This questionnaire included demographic data and information on lymphedema caused by breast cancer treatment and risk reduction practices/ prevention. After twenty minutes, the nurses submitted their completed forms. The participants then received a structured teaching session of 40 minutes on breast cancer-related lymphedema, including its definition, pathophysiology, risk factors, complications, risk reduction practices/ prevention, and management.

Table 1 illustrates the prevention interventions and precautions.

**Table 1:** Prevention Interventions and Precautions* *National Lymphedema Network (2009).  Lymphedema Risk Reduction Practices [21].

Prevention Interventions	Strategies and Precautions
Avoidance of trauma and injury to the arm	The affected arm should not be used for venipuncture, blood pressure monitoring, or injections. Additionally, avoid getting bitten by a bug or scratched by an animal.
Prevention of infection	Providing prompt first aid, spotting and treating any signs of infection right away, avoiding heat and sunlight, taking meticulous care of one's skin and nails, and avoiding paper cuts are all ways to prevent infection.
Avoidance of Arm Constriction	On the affected extremities, stay away from tight clothing and jewelry; wear a padded bra strap to relieve pressure; and refrain from carrying a shoulder bag.
Use and Exercise of the Limb	Use the affected limb sparingly, do not lift anything heavy, and steer clear of repetitive activity.

After the education session, the nurses were given the same questionnaire on breast cancer-related lymphedema. Later, the completed forms were analyzed.

Data analysis

Both descriptive and inferential statistics were used. Distribution, percentage, mean, median, and standard deviation were used in descriptive statistics. SPSS (SPSS Inc. Released 2007. SPSS for Windows, Version 16.0. Chicago, SPSS Inc.) was used to analyze the data. A paired t-test was used to analyze the data, and the p-value of <0.001 levels was highly significant.

## Results

Major findings of the study

Fifty-seven percent (57%) of the subjects had 'moderately adequate' knowledge and 29% had 'highly adequate' knowledge of BCRL risk reduction/prevention practices. Seventy-three percent (73%) of the subjects agreed that written information on BCRL risk reduction/prevention should be provided to the patients. Eighty-five percent (85%) of the subjects accepted that the initial days of breast cancer diagnosis are the best time to provide information on BCRL risk reduction/prevention. Eighty-nine percent (89%) agreed on the measures to improve the dissemination of reducing the risk of lymphedema to breast cancer patients.

Table 2 shows that the pre-test knowledge of information on BCRL risk reduction/prevention education to the patients among the nurses was low at 3.60, which increased to 4.81 (post-test) session, indicating that they gained knowledge regarding the patient education information related to BCRL.

**Table 2:** Knowledge level of BCRL risk reduction/prevention among the nurses Agree Disagree Scale

	Pre Test Score	Post Test Score
Avoid repetitive activities such as pushing or pulling	4.14	4.29
Moisturize every day to prevent infection	4.17	4.23
Do not participate in complimentary therapies.	3.87	3.85
Do not have a tattoo on your affected side	4.52	4.52
Avoid acupuncture on the affected side	4.58	4.72
Avoid any form of venepuncture/injections on the affected side	4.63	4.75
Do not have blood pressure monitoring on your affected side	4.63	4.75
Avoid extreme temperatures	4.31	4.43
Avoid hot tubs	4.15	4.37
Do not use a sauna	3.61	3.94
Exercise 5 times a week for 30 mins	3.9	4.27
Exercise daily with recommended exercises	4.66	4.66
Reduce weight to a BMI of 25 or less	4.13	4.37
Avoid underwire bras	4.07	4.25
Treat any fungal infections promptly	4.54	4.76
Protect your skin from scratches, cuts and burns	4.59	4.81
Wear gloves whilst gardening to prevent infections	4.47	4.67
Use insect repellent whilst on holidays	4.14	4.04
Do not wear any tight jewelry or clothing on your affected side	4.44	4.71
You must wear a garment whilst going on an airplane	4.26	4.27
Do not do any extreme sports	4.30	4.49
Do not sunbathe	4.28	4.51
Do not smoke	4.43	4.75
Do not take dogs for a walk in case they pull your affected side	4.04	4.44
Do not lift over 2 kg in weight on your affected side	3.60	4.36
Do not carry your bag on your affected side	4.04	4.37

Table 3 shows that in the pre-test 41% of respondents had inadequate knowledge of risk reduction and prevention of BCRL, while 21% of respondents had adequate knowledge. However, in the post-test, the proportion of respondents with inadequate knowledge was reduced to 33%. Moreover, the proportion of respondents with adequate knowledge increased to 30%. 

**Table 3:** Knowledge scores amongst nursing staff (N=84)

S.No	Score	Level of knowledge	Pre-test		Post-test	
			Frequency	%	Frequency	%
1	Less than 50	Inadequate	34	41	28	33
2	51 – 75	Moderately Adequate	32	38	31	37
3	Above 75	Adequate	18	21	25	30
Total score:			0-100		0-100	

Table 4 indicates a significant difference between the pre and post-test teaching sessions on knowledge regarding risk reduction/prevention of BCRL, evidenced by a high significant p-value (p < 0.001).

**Table 4:** Pre and post-test level of knowledge regarding risk reduction and prevention of BCRL among nurses

	Mean	SD	Standard Error Mean	t-statistic	df	p-value
Pre-test	4.286	0.97	0.0418	4.237	83	< .001
Post-test	4.452	1.511	0.0639			

Table 5 shows that the best way to disseminate information regarding BCRL risk reduction/prevention is by allowing patients to self-refer to nearby lymphedema clinics and mandatorily measure the arm circumference preoperatively, evidenced by a pre-test score of 4.51, increased to a post-test score of 4.54, respectively.

**Table 5:** Measures to improve the dissemination of information to patients regarding BCRL risk reduction/prevention Agree Disagree Scale

	Pre Test Score	Post Test Score
Have dedicated staff to offer one-to-one sessions on reducing the risk of lymphedema to all breast cancer patients	4.21	4.28
Have dedicated staff to offer to reduce the risk of lymphedema group sessions to all breast cancer patients	4.23	4.38
Development of national 'reducing the risk of lymphedema' leaflets	4.23	4.36
Development of video prescriptions/apps in reducing the risk of lymphedema	4.24	4.30
Development of a self-measurement guide to check the size of arms	4.41	4.61
Make taking arm measurements preoperatively mandatory	4.50	4.54
Allow all breast cancer patients to self-refer to the nearest lymphedema clinic if they have symptoms	4.51	4.54

## Discussion

Nurses play a key role in health promotion and maintenance and are crucial in implementing learning to practice. Relevant care for BCRL patients will require education and training. Patients' chance of acquiring lymphedema can be decreased, and those who already have it can prevent it from getting worse by being informed about it.

Fifty-eight percent of the subjects in this study revealed that the nurses' time to educate the breast cancer survivors on lymphedema prevention and risk reduction was less than an hour per day. In general, nurses state that despite their primary role to educate the patients on preventive aspects, they cannot do so due to an increased number of patients, lack of time, lack of continuing education to update the information, and lack of expectations on patient education. In addition, nurses' inefficient time management, turnover, burnout, lack of appropriate clinical setting, and inadequate nurse-patient ratio are some issues affecting patient education. Even though the effect of BCRL is vast, it has been undiagnosed and neglected for various reasons [6]. Yildirim et al.'s study of ten nurses working in the surgical department found that nurses' knowledge of LE is updated, supporting this behavior of nurses. However, they need to be motivated to organize, schedule, and continually receive training in order to increase LE preventative behaviors in patients [26].

In the current study, 57% of nurses had good knowledge of risk reduction/prevention of BCRL, and 29% had excellent knowledge about BCRL risk reduction/prevention aspects, which is corroborated with the correct mention of aspects for BC patients to reduce risk of lymphedema development. One study highlights that when nurses know the definition, causes, signs and symptoms, physiology, and treatment, it enables early assessment of BCRL risk and recognition of symptoms [27]. 

Patients unaware of BCRL and its symptoms cannot report it at the right time. Education and providing information to patients will help them to reduce the risk and prevent the progression of swelling. In this study, 85% of nursing staff agreed that the best time to provide information on lymphedema risk reduction/prevention is the day of breast cancer diagnosis. Ridner et al., in their study, emphasized that education on BCRL pre-treatment will enable the recollection of lymphedema information in breast cancer survivors and might have an impact on reducing the risk of developing lymphedema [6].

In the present study, 96% of nursing staff agreed to measure the weight of the breast cancer survivors, and 73% agreed to calculate body mass index (BMI) as they were aware of the close relationship between BMI and lymphedema development. High BMI during breast cancer diagnosis is a well-established risk factor for developing BCRL [14]. This finding is supported by Ridner and colleagues' smaller prospective study using perometry in which they found that patients with a BMI of 30 kg/m2 or above were 3.6 times more likely to develop lymphedema (95% CI: 1.42-9.04; P=0.007) [28]. 

Table 2 shows that the pre-test knowledge of information on BCRL risk reduction/ prevention education to the patients among the nurses was as low as 3.60, increasing to 4.81 post-test, indicating that they gained knowledge regarding the patient education information related to BCRL. This is due to the fact that, despite receiving training on LE during their education, they are not confident that they possess a command of the subject because they do not receive ongoing in-service training on this condition. Therefore, effective care depends on continuing education and knowledge, skills, and willingness among the nurses.

As per breast cancer survivors and the nursing personnel, information on lymphedema risk reduction and prevention needs to be provided at various timeframes from breast cancer diagnosis during treatment through follow-up and beyond. 

Table 5 shows that the best way to disseminate information to patients regarding BCRL risk reduction/prevention is allowing patients to self-refer to nearby lymphedema clinics and measuring the arm circumference preoperatively mandatory, evidenced by a pre-test score of 4.51, increased to a post-test score of 4.54, respectively. In this study, nurses were confident that providing adequate information on lymphedema risk reduction and prevention on time may help breast cancer survivors prevent lymphedema's development. Sherman et al. have appropriately narrated in their study that information provided on lymphedema by the nursing staff and written information through brochures or booklets played a vital role in lymphedema risk reduction [28].

Nurses are the key personnel in the healthcare team, who play a vital role in health promotion and maintenance and are crucial in implementing learning to practice. The healthcare delivery system in the present scenario needs to emphasize the preventive and rehabilitative aspects of patients with breast cancer and secondary lymphedema. Creating awareness among healthcare professionals, utilizing it for breast cancer patients, and involving family members will help control the severity of the condition.

This study motivates students and oncology staff nurses to practice pre-habilitation by assessing baseline data like arm measurements preoperatively and preoperative education on BCRL to prevent and reduce the risk of breast cancer-related lymphedema development.

The study has some limitations; one is the small sample size; hence the findings cannot be generalized. Secondly, the study was conducted among nursing personnel in a single hospital. The outcome variables may vary depending on the educational background and specialization of the participants, hospital policy, and study locality. The study can be replicated with a large sample size of different healthcare professionals to create and reinforce awareness of prevention and risk reduction practices among post-mastectomy patients. The study can be conducted as an experimental research design with interventions and follow-up of the patients to assess the adherence to self-care practices compared with the study and control group.

## Conclusions

The study results show increased knowledge of BCRL risk reduction practices in the post-educational session compared to pre-test data among the nurses. To lower the risk for lymphedema, nurses should educate breast cancer patients about their risk, offer instructions, and promote self-care practices.

According to this study, a structured teaching/education program for nurses regarding the risk of LE incidence is urgently needed, primarily to provide information for patients who have undergone breast cancer surgery. Nurses will need to transition from past practices to the latest developments in rehabilitation with new cancer patients. Hence regular in-service education is required to update the present knowledge. 
